# Growth performance, digestive function, thyroid activity, and immunity of growing rabbits fed olive cake with or without *Saccharomyces cerevisiae* or citric acid

**DOI:** 10.1007/s11250-023-03794-y

**Published:** 2023-10-25

**Authors:** Ahmed M. Elbaz, Bahaa Farrag, Noura M. Mesalam, Hamdy A. Basuony, Aml M. M. Badran, Abdel-Moneim Eid Abdel-Moneim

**Affiliations:** 1 Desert Research Center, Mataria, Cairo, Egypt; 2 Biological Applications Department, Nuclear Research Center, Egyptian Atomic Energy Authority, Cairo, Egypt; 3 Poultry Breeding Department, Animal Production Research Institute, Agricultural Research Center, Ministry of Agriculture, Dokki, Giza, Egypt

**Keywords:** Performance, Digestive function, Antioxidant status, Immunity, Rabbits

## Abstract

The present study investigated the impact of dietary inclusion of olive cakes (OC) with or without *Saccharomyces cerevisiae* (SC) and citric acid (CA) on growth, digestive function, thyroid activity, antioxidant status, immunity, and intestinal architecture of growing rabbits. One hundred forty 35-day-old male New Zealand white rabbits were randomly assigned into seven experimental groups with five replicates each, as follows: control (CN), fed the basal diet; OC20 and OC25, fed diets with 20 and 25% OC; OS20 and OS25, fed diets containing 20 and 25% OC with *S. cerevisiae* at 5 g/kg diet; OA20 and OA25, fed diets supplemented with 20 and 25% OC with 1.0% citric acid. No differences in live body weight, feed intake, feed conversion ratio, and carcass traits were noticed among experimental groups, while body weight gain and carcass (%) were increased (*P* < 0.05) in OS20 compared to the control. Digestibility coefficients of all nutrients and activities of amylase, cellulose, and trypsin did not differ in treated groups compared to the control except for OS20, which recorded enhancement in nutrient digestibility. Plasma triiodothyronine and thyroxine were elevated (*P* < 0.05), while triglycerides and cholesterol were reduced (*P* < 0.05) in OS20 compared to CN. Plasma concentrations of immunoglobulin M and G and superoxide dismutase were increased in treated groups compared to the control. Dietary inclusion of SC and CA improved rabbits’ intestinal health, as the cecal *Lactobacillus* count was increased, *E. coli* count was decreased, and villus height was elevated in SC- and CA-treated groups. In conclusion, dietary incorporation of SC or CA enhanced the nutritional value of OC and improved growth performance, nutrient digestibility, thyroid activity, antioxidative status, and gut health of growing rabbits.

## Introduction

Continuous increases in the prices of conventional feed materials force nutritionists to search for unconventional feed alternatives such as agricultural residues and agricultural by-products (Elbaz 2021; Abdel-Moneim et al. 2020a; Ibrahim et al. 2020; Abd El-Moneim and Sabic 2019), while the use of unconventional feed materials in feeding monogastric animals is hindered due to their content of anti-nutritional materials, which hinder the utilization of some nutrients, and thus negatively affect their performance (Saleh et al. 2022). Olive cake (OC) is a by-product of the olive oil extraction process, representing about 35–40% of the weight of pressed olive fruit. The olive cake is an agricultural waste that is not efficiently utilized and is considered a source of environmental pollution (Elbaz et al. 2020). Additionally, OC contains some anti-nutritional substances, such as tannins, that negatively affect nutrient digestibility, reduce the activity of digestive enzymes, and eliminate the absorption of amino acids, minerals, and sugars (Kim and Miller 2005). Fortunately, dietary incorporation of some feed additives, such as probiotics or organic acids, or the chemical treatments of agricultural by-products, such as sodium bicarbonate, can reduce these negative effects of anti-nutritional factors (Abdel-Moneim et al. 2021; Rowghani et al. 2008). Therefore, further investigations are required to evaluate the most efficient treatment method for the agricultural by-products in order to maximize their use in rabbit feed.

From another perspective, growing rabbits suffer from epidemiological intestinal problems and digestive disorders during the weaning period due to their inability to modify the intestinal microbes, eventually leading to high mortality rate (Bivolarski and Vachkova 2014). However, incorporating fibrous feedstuffs and probiotics in growing rabbits’ diets can regulate gut movement and modify the intestinal microbiome (Elbaz et al. 2021; Gidenne 2015). *Saccharomyces cerevisiae* is one of the most effective and commonly used probiotics in poultry and rabbit feed. *S. cerevisiae* produces several nutrients (i.e., vitamins, volatile fatty acids, amino acids, and exogenous enzymes, i.e., lipases, proteases, and amylases) that suppress enteric pathogens and the detoxification of pathogenic toxins, modulate gut microbes, and enhance immune response (Abd El‐Hack et al. 2020). Furthermore, the cell wall of *S. cerevisiae* is considered a potent prebiotic since it consists of *α*-mannan, mannoproteins, *β*-glucan, and chitin, which harbor numerous proteins that play a crucial role in molecular recognition and adhesion (Abd El-Hack et al. 2021; Ahiwe et al. 2021). Organic acids, such as citric acid, have been classified as an antibiotic alternative due to their antimicrobial and growth-promoting activities. Organic acid supplementation to monogastric animals can amend gut homeostasis, improve the intestine architecture, lower gut pH, elevate the enumeration of beneficial bacteria, and consequently improve their performance (Elbaz et al. 2021).

Limited studies have been conducted to evaluate the efficacy of the biological treatment of OC and its impacts when supplemented in rabbits’ diets. Therefore, the present study evaluated the effect of incorporating OC into rabbit feed with or without *S. cerevisiae* or citric acid on performance, digestibility, humoral immunity, antioxidative status, ileal histomorphometry, and microbial enumeration of growing rabbits.

## Materials and methods

### Animals and diets

One hundred forty 35-day-old male New Zealand white rabbits, with an average initial weight of 637.8 ± 10.4 g, were randomly assigned into seven experimental groups with five replicates each, as follows: control (CN), fed the control diet; OC20 and OC25, fed diets with 20 and 25% olive cake; OS20 and OS25, fed diets containing 20 and 25% olive cake with *Saccharomyces cerevisiae* at 5 g/kg diet; OA20 and OA25, fed diets supplemented with 20 and 25% olive cake with 1.0% citric acid. The experimental diets were formulated to meet the nutritional requirements of growing rabbits according to the recommendations of NRC (1977), as shown in Table 1. Clean fresh water and a pelleted diet were offered ad libitum. The rabbits were housed under uniform management conditions in a well-ventilated room and the temperature during the experiment was from 20 to 25 °C.

**Table 1:** Ingredients and chemical composition of experimental diets

Ingredients	CN	OC20	OC25
Olive cake	0.000	20.00	25.00
Yellow corn	15.00	10.50	9.60
Soybean meal (44% CP)	17.50	20.00	21.20
Sunflower meal	5.00	5.50	5.50
Alfalfa dehydrated	39.50	20.30	15.00
Barley	9.00	9.00	9.00
Wheat bran	10.00	10.00	10.00
Limestone	0.50	1.00	1.00
Di-calcium phosphate	0.70	0.90	0.90
Premix (vit-min)^a^	0.50	0.50	0.50
Salt	0.30	0.30	0.30
Molasses	2.00	2.00	2.00
Calculated analysis (%)			
Dry matter	88.35	88.74	88.81
Crude protein	18.48	18.35	18.42
Ether extract	2.35	2.51	2.49
Crude fiber	13.81	14.62	15.07
Calcium	1.17	1.18	1.17
Phosphorus	0.55	0.56	0.54
Metabolizable energy (MJ/kg)	10.46	10.37	10.32
Proximate composition of the olive cake (%)			
Dry matter	90.6		
Crude protein	6.8		
Crude fiber	38.2		
Crude fat	7.7		
Nitrogen-free extract	32.5		
Metabolizable energy (MJ/kg)	9.54		

^a^Each 1 kg of vitamins and minerals premix contained: vitamin A, 10 000 IU; vitamin D3, 1800 UI; vitamin E, 15 mg; vitamin K3, 4.5 mg; vitamin B1, 0.5 mg; vitamin B2, 4 mg; vitamin B12, 0.001 mg; folic acid, 0.1 mg; pantothenic acid, 7 mg; nicotinic acid, 20 mg; I, 1 mg; Mn, 60 mg; Cu, 5.5 mg; Zn, 75 mg; Fe, 40 mg; Co, 0.3 mg; Se, 0.1 mg; robenidine, 52.8 mg

Citric acid was purchased from Weifang Ensign Industry Co. Ltd., Shandong, China, while *Saccharomyces cerevisiae* was purchased from a commercial store and analyzed. The chemical composition of *S. cerevisiae* was 92.6% dry matter, 42.8% crude protein, 2.7% crude fiber, and 1.3% crude fat.

### Chemical analysis of olive cake

OC was obtained from a commercial olive oil mill, grounded, sun-dried, and analyzed in the regional laboratory for food and feed in Giza, Egypt (AOAC 2000), as described by Abd El-Moneim and Sabic (2019). The chemical composition of OC is presented in Table 1.

### Growth performance

At 56 and 77 days of age, the live body weight (LBW), body weight gain (BWG), feed intake (FI), and daily mortality were recorded. Feed conversion ratio (FCR) was calculated (FI divided by BWG). At the end of the experiment (77 days of age), five rabbits were randomly selected from each experimental group, individually weighed and slaughtered for carcass evaluation. Carcass, liver, heart, kidney, lungs, and giblets were weighed and their relative weights to LBW were calculated.

### Nutrient digestibility

At the end of the experimental period, five rabbits/group were housed in metabolic cages and kept to adapt for 24 h before the collection period. Feces were collected for 3 days, dried (65 °C for 48 h), grounded, and stored in polyethylene bags (− 20 °C) until the chemical analyses. Samples of feces and feed were analyzed for dry matter, crude protein, crude fiber, ether extract, and nitrogen-free extract following AOAC (2000) methods.

### Digestive enzymes activity

During slaughtering, digesta samples were taken from the duodenum to estimate the activity of digestive enzymes. Activities of cellulase, trypsin, and amylase were determined using specialized commercial kits (Nanjing Jiancheng Bioengineering Institute, China) as described by Abdel-Moneim et al. (2020b).

### Blood biochemical analysis

At slaughtering, blood samples were collected in anticoagulant tubes, centrifuged directly at 3000 × g for 15 min, and plasma samples were collected and stored at − 20 °C. Total protein, albumin, cholesterol, triglycerides, high-density lipoprotein (HDL), low-density lipoprotein (LDL), glucose, aspartate aminotransferase (AST), and alanine aminotransferase (ALT) levels were determined colorimetrically (Spectronic 1201, Milton Roy, Ivyland, PA, USA) following the manufacturer’s instructions (Spinreact Co., Girona, Spain). Triiodothyronine (T3) and thyroxine (T4) were assayed using radioimmunoassay (RIA) kits (Ibrahim et al. 2020).

### Antioxidant activity

The activities of malondialdehyde (MDA), superoxide dismutase (SOD), and glutathione peroxidase (GPx) were measured as an indicator of oxidation state according to the manufacturer’s instructions using commercial kits (Bio diagnostic company, Egypt).

### Humoral immunity

Plasma concentrations of circulating immunoglobulin IgA, IgM, and IgG were estimated using immunoglobulin ELISA quantitation kits (Life Diagnostics Inc., PA, USA).

### Ileal histomorphometry

For histological examination, the length of the intestine was measured, and samples were taken from the ileum (3 cm of tissue samples). Collected samples were rinsed well with saline solution (0.85% NaCl) and kept in formalin solution (10%) until morphometric analysis was performed. Samples were cut to 4 cm and then stained with hematoxylin and eosin on specially designed slides (Shehata et al. 2022). The slides were examined through a light microscope equipped with a computed digital camera (Labomed, LX 400. Labo America, Inc. USA) to measure the villi height and crypt depth, and the villus height:crypt depth ratio was calculated.

### Microbial enumeration

At slaughtering, digesta samples were collected from the cecum of five rabbits/group, placed in sterile bags, and kept at − 20 °C until microbial counts. Samples were diluted (10^−1^ to 10^−7^), immersed in an agar medium suitable for each microbe, and incubated under aerobic or anaerobic conditions and the required temperature as described by Abdel-Moneim et al. (2022c). Total bacterial count, *Escherichia coli*, and *Lactobacillus* bacteria were enumerated (DeMan agar, MacConkey agar, and MRS agar, respectively).

### Statistical analysis

The SPSS software’s general linear model (GLM) approach evaluated the collected data in a completely randomized design. Shapiro–Wilk and Levene tests were used to test the normal distribution of data as well as the homogeneity of variance. The statistical model for the ANOVA was as follows:** Y**_**ij**_ = **µ** + **T**_**i**_ + **£**_**ij**_, where **Y**_**ij**_ = observation, **µ** = overall mean, **T**_**i**_ = a fixed effect of treatments, and **£**_**ijk**_ = experimental error. Tukey’s test was used to determine the significance of mean differences, and all differences were judged significant at *P* > 0.05.

## Results

### Growth performance

LBW, BWG, FI, and FCR of growing rabbits fed OC with or without *S. cerevisiae*, or citric acid are shown in Table 2. During the periods from 35 to 56 and 57 to 77 days, no differences in LBW, FI, and FCR were noticed among experimental groups, while BWG was increased (*P* < 0.05) in OS20 (35–56 days) and OS20, A20, and OS25 (57–77 days) compared to the control. During the overall period, BWG was improved (*P* < 0.05) in OS20 and reduced in OC25 compared to CN. The overall FCR was not affected among experimental groups except for OC25, which was elevated (*P* < 0.05) compared to the control.

**Table 2:** Effect of dietary level of inclusion of olive cake (OC) with or without Saccharomyces cerevisiae or citric acid on growth performance of growing rabbits

Item	CN	OC20	OS20	OA20	OC25	OS25	OA25	SEM	*P*-value
Initial body weight	641.4	639.1	633.5	631.6	634.2	648.3	636.9	19.5	0.652
Period 35–56 d									
Live body weight at 56d	1082	1062	1095	1076	1034	1074	1068	6.18	0.234
Body weight gain (g/d)	22.0^b^	21.2^b^	23.1^a^	22.2^b^	20.0^c^	21.3^b^	21.6^b^	1.87	0.038
Daily feed intake (g/d)	34.8	35.2	35.7	35.5	34.8	35.1	35.6	1.51	0.411
Feed conversion ratio (g/g)	1.96	2.04	1.93	1.98	2.03	1.97	1.99	0.09	0.170
Period 57–77d									
Live body weight at 75 d	1593	1576	1638	1604	1539	1596	1575	9.07	0.059
Body weight gain (g/d)	25.6^c^	25.7^c^	27.1^a^	26.5^b^	25.2^c^	26.1^b^	25.4^c^	2.03	0.001
Daily feed intake (g/d)	56.5	57.7	58.9	58.3	57.4	57.5	56.7	1.26	0.728
Feed conversion ratio (g/g)	2.21	2.25	2.18	2.20	2.28	2.20	2.23	1.10	0.061
Period 36–77d									
Body weight gain (g/d)	23.8^b^	23.3^bc^	25.1^a^	24.3^b^	22.7^c^	23.9^b^	23.2^bc^	1.53	0.033
Daily feed intake (g/d)	91.1	92.8	94.3	93.8	92.0	92.7	92.3	2.27	0.874
Feed conversion ratio (g/g)	3.82^bc^	3.97^ab^	3.74^c^	3.86^b^	4.04^a^	3.88^b^	3.96^ab^	0.21	0.014

^a^^,b,c^Means in the same row with different superscripts are significantly different (*P* < 0.05); *SEM*, standard error of means; *CN*, control diet; *OC20* and *OC25*, fed diets with 20 and 25% OC; *OS20* and *OS25*, fed diets containing 20 and 25% OC with *S. cerevisiae* at 5 g/kg diet; *OA20* and OA25, fed diets supplemented with 20 and 25% OC with 1.0% citric acid

Dietary incorporation of both levels of OC with or without *S. cerevisiae* or citric acid did not affect the relative weights of the liver, kidney, heart, lungs, giblets, and total edible parts (Table 3). Carcass percentage was increased significantly in OS20 and numerically in the remaining groups except for OC25 compared to CN.

**Table 3:** Effect of dietary level of inclusion of olive cake with or without *Saccharomyces cerevisiae* or citric acid on carcass traits of growing rabbits

Item	CN	OC20	OS20	OA20	OC25	OS25	OA25	SEM	*P*-value
Live body weight (g)	1603	1580	1632	1607	1536	1604	1581	11.58	0.074
Carcass (%)	56.4^b^	56.7^b^	63.2^a^	59.4^ab^	55.6^b^	60.7^ab^	57.6^b^	7.012	0.031
Liver (%)	3.21	3.16	3.08	3.23	3.37	3.15	2.98	0.370	0.915
Kidney (%)	0.67	0.65	0.68	0.70	0.68	0.66	0.67	0.082	0.112
Heart (%)	0.32	0.28	0.30	0.33	0.31	0.32	0.30	1.031	0.207
Lungs (%)	0.67	0.71	0.69	0.74	0.68	0.74	0.62	0.079	0.095
Giblets (%)	4.70	4.54	4.93	4.85	4.71	4.32	4.61	0.025	0.320
Total edible parts (%)	60.58	59.76	60.81	61.27	59.53	60.21	60.34	3.741	0.191

^a^^,b^Means in the same row with different superscripts are significantly different (*P* < 0.05); *SEM*, standard error of means; *CN*, control diet; *OC20* and *OC25*, fed diets with 20 and 25% OC; *OS20* and *OS25*, fed diets containing 20 and 25% OC with *S. cerevisiae* at 5 g/kg diet; *OA20* and *OA25*, fed diets supplemented with 20 and 25% OC with 1.0% citric acid

### Nutrient digestibility and digestive enzymes activity

Dietary inclusion of both levels of OC did not affect the digestibility coefficients of dry matter, crude protein, crude fiber, ether extract, and nitrogen-free extract (Table 4). However, supplementation of *S. cerevisiae* improved (*P* < 0.05) all nutrient digestibility in OS20 and dry matter and crude protein digestibility in OS25 compared to CN. Citric acid inclusion in rabbits’ diets did not significantly enhance nutrient digestibility except for crude protein digestibility, which was increased (*P* > 0.01) in OA20. Dietary inclusion of OC with or without *S. cerevisiae* and citric acid did not affect the activities of amylase, cellulase, and trypsin compared to the control.

**Table 4:** Effect of dietary level of inclusion of olive cake (OC) with or without *Saccharomyces cerevisiae* or citric acid on digestibility coefficients and digestive enzymes activity of growing rabbits

Item	CN	OC20	OS20	OA20	OC25	OS25	OA25	SEM	*P*-value
Digestibility coefficients (%)									
Dry matter	62.5^b^	62.7^b^	65.6^a^	63.1^b^	62.0^b^	64.8^a^	62.4^b^	1.270	0.021
Crude protein	70.2^b^	69.4^b^	71.8^a^	71.2^a^	68.8^b^	72.1^a^	70.6^b^	2.033	0.006
Crude fiber	43.3^b^	41.5^bc^	46.6^a^	44.7^ab^	40.9^c^	45.2^ab^	43.7^b^	0.547	0.014
Ether extract	82.1^b^	82.7^b^	85.4^a^	84.3^ab^	81.6^bc^	84.5^ab^	82.9^b^	4.081	0.027
Nitrogen-free extract	56.5^b^	51.0^c^	58.2^a^	57.1^b^	49.7^c^	57.6^b^	55.8^b^	1.994	0.001
Digestive enzymes									
Amylase (U/g)	3.31	2.94	3.42	3.28	3.06	3.38	3.19	1.091	0.062
Cellulase (U/g)	18.2	17.6	18.5	18.3	18.1	19.4	18.6	0.843	0.229
Trypsin (KU/mg)	2.06	2.11	2.17	2.24	2.03	2.15	2.27	0.231	0.105

^a^^,b,c^Means in the same row with different superscripts are significantly different (*P* < 0.05); *SEM*, standard error of means; *CN*, control diet; *OC20* and *OC25*, fed diets with 20 and 25% OC; *OS20* and *OS25*, fed diets containing 20 and 25% OC with *S. cerevisiae* at 5 g/kg diet; *OA20* and *OA25*, fed diets supplemented with 20 and 25% OC with 1.0% citric acid

### Plasma biochemical indices

Plasma biochemical parameters of growing rabbits affected by different experimental diets were estimated as shown in Tables 5 and 6. Plasma concentrations of glucose, HDL, LDL, total protein, albumin, globulin, AST, and ALT were not altered among the treatment groups compared to the control. Plasma triglycerides and cholesterol levels were reduced (*P* < 0.05) in OS20 and elevated in OC25 compared to CN and the remaining treated groups. T3 and T4 levels were significantly (*P* < 0.05) elevated in rabbits fed 20% OC supplemented with *Saccharomyces cerevisiae* (SC) and numerically in the rest of the groups compared to CN, OC20, and OC25 groups.

**Table 5:** Effect of dietary level of inclusion of olive cake (OC) with or without *Saccharomyces cerevisiae* or citric acid on blood biochemical profile of growing rabbits

Item	CN	OC20	OS20	OA20	OC25	OS25	OA25	SEM	*P*-value
Glucose (mg/dL)	109	114	112	116	113	107	111	3.181	0.205
Triglycerides (mg/dL)	66.1^b^	68.5^ab^	61.6^c^	65.3^b^	71.7^a^	63.1^bc^	66.8^b^	4.092	0.018
Cholesterol (mg/dL)	76.9^b^	81.6^a^	71.5^c^	74.2^bc^	82.4^a^	73.7^bc^	76.3^b^	2.155	0.032
HDL (mg/dL)	27.5	27.1	28.2	27.9	26.7	27.8	28.4	0.908	0.217
LDL (mg/dL)	19.4	18.7	17.9	18.6	19.1	18.4	19.6	0.027	0.096
Total protein (g/dL)	6.18	6.32	6.27	6.19	6.20	6.34	6.15	0.184	0.334
Albumin (g/dL)	3.62	3.67	3.64	3.59	3.56	3.67	3.60	0.107	0.122
Globulin (g/dL)	2.57	2.65	2.63	2.61	2.64	2.68	2.54	0.094	0.407

^a^^,b,c^Means in the same row with different superscripts are significantly different (*P* < 0.05); *SEM*, standard error of means; *HDL*, high-density lipoprotein cholesterol; *LDL*, low-density lipoprotein cholesterol; *CN*, control diet; *OC20* and *OC25*, fed diets with 20 and 25% OC; *OS20* and *OS25*, fed diets containing 20 and 25% OC with *S. cerevisiae* at 5 g/kg diet; *OA20* and *OA25*, fed diets supplemented with 20 and 25% OC with 1.0% citric acid

**Table 6:** Effect of dietary level of inclusion of olive cake (OC) with or without *Saccharomyces cerevisiae* or citric acid on hepatic enzyme and thyroid hormone activities of growing rabbits

Item	CN	OC20	OS20	OA20	OC25	OS25	OA25	SEM	*P*-value
AST (U/L)	49.1	52.4	48.9	50.7	51.8	50.3	49.7	1.116	0.185
ALT (U/L)	15.8	16.2	15.6	15.3	16.5	15.8	16.1	0.691	0.073
T3 (nmol/L)	1.94^b^	1.88^b^	2.43^a^	2.27^ab^	1.91^b^	2.23^ab^	2.06^b^	4.220	0.019
T4 (nmol/L)	34.7^b^	33.6^b^	41.1^a^	37.8^b^	34.6^b^	38.9^ab^	36.3^b^	1.931	0.021
T3/T4 ratio	0.055	0.056	0.058	0.059	0.054	0.056	0.057	0.040	0.312

^a,b^Means in the same row with different superscripts are significantly different (*P* < 0.05); *SEM*, standard error of means; *AST*, aspartate aminotransferase; *ALT*, alanine aminotransferase; *T3*, triiodothyronine; *T4*, thyroxine; *CN*, control diet; *OC20* and *OC25*, fed diets with 20 and 25% OC; *OS20* and *OS25*, fed diets containing 20 and 25% OC with *S. cerevisiae* at 5 g/kg diet; *OA20* and *OA25*, fed diets supplemented with 20 and 25% OC with 1.0% citric acid

### Antioxidant activity

As presented in Table 7, the dietary supplements increased (*P* < 0.01) SOD activity, while the activity of MDA and GPx was not affected. The highest value of SOD was recorded on OS20, followed by OS25.

**Table 7:** Effect of dietary level of inclusion of olive cake with (OC) or without *Saccharomyces cerevisiae* or citric acid on immunity and antioxidant activity of growing rabbits

Item	CN	OC20	OS20	OA20	OC25	OS25	OA25	SEM	*P*-value
Immunoglobulin									
IgA (ng/mL)	29.4	28.7	30.5	29.6	28.4	29.2	29.8	0.927	0.095
IgM (ng/mL)	24.9^c^	23.1^c^	31.4^ab^	33.7^a^	24.6^c^	33.4^a^	30.2^b^	3.112	0.001
IgG (ng/mL)	22.1^b^	21.8^b^	26.0^a^	24.2^ab^	21.5^b^	25.7^a^	23.6^ab^	1.005	0.016
Antioxidant activity									
MDA (nmol/mL)	1.57	1.46	1.53	1.58	1.49	1.52	1.56	0.554	0.182
SOD (U/mL)	31.4^c^	32.5^bc^	37.9^a^	34.5^b^	33.7^b^	36.2^ab^	34.1^b^	6.243	0.004
GPx (U/L)	26.2	27.0	26.8	25.7	26.5	27.2	26.9	2.099	0.243

^a,b,c^Means in the same row with different superscripts are significantly different (*P* < 0.05); *SEM*, standard error of means; *MDA*, malondialdehyde; *SOD*, superoxide dismutase; *GPx*, glutathione peroxidase; *CN*, control diet; *OC20* and *OC25*, fed diets with 20 and 25% OC; *OS20* and *OS25*, fed diets containing 20 and 25% OC with *S. cerevisiae* at 5 g/kg diet; *OA20* and *OA25*, fed diets supplemented with 20 and 25% OC with 1.0% citric acid

### Humoral immunity

The effects of OC incorporation, with or without *S. cerevisiae* or citric acid, in growing rabbits’ diets on humoral immunity are shown in Table 7. The results revealed a noticeable immune improvement in the growing rabbits fed OC with SC or citric acid (CA), as the concentrations of the plasma immunoglobulins IgM and IgG were elevated in OS20, OS25, OA20, and OA25 compared to CN, OC20, and OC25. IgA concentration was not altered among experimental groups.

### Ileal histomorphometry

Dietary supplementation of SC or CA to OC in the experimental diets increased (*P* < 0.01) the villus height (VH) in OS20, OA20, and OS25 compared to the unsupplemented group (Table 8). The dietary supplements did not affect the small intestine length and crypt depth (CD). VH:CD ratio was increased (*P* < 0.01) in OS20, OA20, and OS25 compared to CN. The highest VH value was recorded in OS20 followed by OS25 and OA20.

**Table 8:** Effect of dietary level of inclusion of olive cake (OC) with or without *Saccharomyces cerevisiae* or citric acid on ileum histomorphometry and cecum microbial populations (Log^10^ CFU g^−1^) of growing rabbits

Item	CN	OC20	OS20	OA20	OC25	OS25	OA25	SEM	*P*-value
Histomorphometry									
Small intestine length (cm)	162.4	158.2	160.1	156.7	160.4	161.5	158.6	2.018	0.781
Villus height, (VH, μm)	401^b^	378^b^	484^a^	453^a^	390^b^	468^a^	428^ab^	5.331	0.001
Crypt depth, (CD, μm)	81.6	80.1	89.3	85.4	82.2	87.3	82.7	3.457	0.094
VH:CD ratio	4.93^b^	4.73^b^	5.40^a^	5.29^a^	4.76^b^	5.35^a^	5.16^ab^	1.091	0.022
Microbial populations									
Total bacterial count	18.6	17.2	17.4	18.2	18.4	19.1	18.6	1.877	0.218
*Lactobacillus*	4.51^c^	4.68^c^	9.30^a^	7.54^b^	4.26^c^	8.05^ab^	7.31^b^	2.513	0.001
*Escherichia coli*	6.22^b^	6.54^b^	3.25^d^	4.39^c^	6.68^b^	3.91^ cd^	4.20^c^	0.922	0.001

^a^^,b,c,d^Means in the same row with different superscripts are significantly different (*P* < 0.05); *SEM*, standard error of means; *CN*, control diet; *OC20* and *OC25*, fed diets with 20 and 25% OC; *OS20* and *OS25*, fed diets containing 20 and 25% OC with *S. cerevisiae* at 5 g/kg diet; *OA20* and *OA25*, fed diets supplemented with 20 and 25% OC with 1.0% citric acid

### Microbial enumeration

Cecal bacteria counts of growing rabbits as affected by dietary incorporation of OC with or without *S. cerevisiae* or citric acid are presented in Table 8. Including *S. cerevisiae* and citric acid to the OC in rabbit feed improved the cecal microbial population. *Lactobacillus* count was increased (*P* < 0.01) in OS20, OS25, OA20, and OA25, while *E. coli* count was decreased (*P* < 0.01) in the same groups compared to the control and OC-supplemented groups. The total bacterial count was not affected by the experimental diet. The highest *Lactobacillus* and lowest *E. coli* counts were recorded in SC-treated groups, followed by CA-treated ones.

## Discussion

The availability of low-cost raw materials with high nutritional value for animal feed is a major target of animal producers and nutritionists. The olive cake is a by-product of the olive oil industry characterized by its economic and high nutritive values (Abd El-Moneim and Sabic 2019; Abd El-Moneim et al. 2019; Elbaz et al. 2020). Results of the current study showed that the inclusion of SC or CA enhanced the nutritional value of OC when added to rabbits’ diets. These results revealed that rabbits fed the different levels of OC (20–25%) with SC or CA were more efficient in feeding utilization than those received OC and control diets without supplements. The results agreed with those reported by Cesari et al. (2008) and Suryanarayana and Suresh (2014), who concluded that SC or CA supplementation in the diet improved body weight gain and the efficiency of feed utilization and enhanced nutrient digestibility. Additionally, Kliševičiūtė et al. (2016) stated that the addition of a mixture of organic acid in rabbit feed increased the body weight of rabbits. As well, Celi et al. (2017) and Shirani et al. (2019) indicated that adding probiotics to growing rabbit feeds improved BWG and FCR. The noticeable improvement in the productive performance in our results may be due to the role of SC or CA in improving the intestinal microbial environment by decreasing the pH value, reducing disease-causing microbes, and decreasing the levels of antinutrients in OC, which improves rabbit health and food utilization efficiency (Abd El-Moneim et al. 2020; Abd El‐Hack et al. 2020; Abdel-Moneim et al. 2020c; Elbaz et al. 2021; Shehata et al. 2021).

Including different levels of OC without supplements did not affect the carcass traits, while there was an increase in the carcass percentage with the addition of SC in OS20 group. The enhancement in carcass weight by the addition of organic acids or probiotics may be due to their role in enhancing nutrient digestibility by modifying the gut microbial content (reduction in pathogenic load) and gut morphology, which leads to increased digestion and absorption of nutrients and enhanced feed conversion ratio (Afsharmanesh et al. 2010; Elbaz et al. 2021). In line with our findings, Mousa and Abd El-Samee (2002) reported that carcass traits of rabbits were not affected by olive pulp inclusion in rabbits’ diets. Reports have shown that diets containing CA or probiotics achieved increased carcass weight in rabbits and poultry (Abdel-Khalek et al. 2012; El-Sawy et al. 2021). However, others reported that the addition of probiotics did not affect the carcass characteristics of rabbits (Ayyat et al. 2018; Bhatt et al. 2017). These discrepancies in the effect of probiotics on carcass traits might be attributed to several factors, including diet composition, probiotics dosage and strain, and animal species (e.g., gender, age, or breed) (Abd El‐Hack et al. 2020).

Our results indicate that nutrient digestibility was improved in rabbits fed 20% OC supplemented with *S. cerevisiae* and citric acid, explaining the improvement in growth performance. Probiotics and organic acids contribute to improving nutrient digestibility by reducing antinutrients (Kishawy et al. 2018) and adjusting gut pH, which stimulates the activity of beneficial microbes and reduces the number of pathogenic microbes, thus contributing to the availability of nutrients (Abd El‐Hack et al. 2020; Kishawy et al. 2018). Our results are consistent with those of Bhatt et al. (2017), who revealed that adding probiotics to growing Chinchilla rabbit diets improved the digestibility of dry matter, crude protein, organic matter, and ether extract. Debi et al. (2010) also reported that the inclusion of citric acid in rabbits’ diets enhanced nutrient digestibility.

Digestive enzyme activities in the present study were not significantly affected by the dietary supplements but were numerically improved. The improvement in digestive enzyme activity could be attributed to the decrease in gut pH, which enhanced the activity of digestive enzymes (Gallois et al. 2005), and the contribution of exoenzymes secreted by probiotic bacteria along with the host’s endogenous enzymes (Wang and Gu 2010). Earlier studies varied about the effectiveness of organic acids or probiotics on the activity of digestive enzymes. In agreement with the results of this study, Rodjan et al. (2018) reported that the probiotic or organic acid did not affect digestive enzyme activities in broilers. However, Abdel-Moneim et al. (2020b) reported that the addition of *Bacillus subtilis* to the quail diet increased the activities of amylase, protease, and lipase. The discrepancy in these results may be due to the level and type of organic acids and probiotics added to the animal diet.

In the present study, biochemical blood metabolites did not differ among experimental groups, except cholesterol and triglycerides levels decreased in OS20 and elevated in OC25. The unaffected plasma protein and liver enzyme activity indicates that the liver was not exposed to stress or damage as a result of the experimental diets in the current study. Lower cholesterol levels in *S. cerevisiae*–treated groups may be due to its ability to incorporate cholesterol into their cellular membrane and convert it into coprostanol, which is directly excreted with the feces, thus reducing blood cholesterol (Aluwong et al. 2013; Elbaz 2021; Ooi and Liong 2010). The results of this study agree with those of Abd El-Moneim and Sabic (2019), who reported that feeding olive pulp with probiotics did not affect biochemical blood indices and reduced serum cholesterol. Moreover, El-Afifi et al. (2001) observed that plasma levels of total protein and globulin were not affected significantly by including olive pulp in rabbit diets containing citric acid. Adil et al. (2010) stated that cholesterol did not significantly differ due to the treatment by organic acids.

Thyroid hormones play an important role in the metabolism of lipids and carbohydrates, maintenance of energy homeostasis, regulation of digestion, and thermoregulation of the body, and affect the activity of the immune system (Williams and Bassett 2018). Many factors affect the activity of thyroid hormones, such as dietary composition, age, ambient temperature, and pathophysiologic status (Lin et al. 2008). Our results showed significant changes in thyroid hormones, where the level of T3 and T4 significantly increased with the addition of SC and CA. These results were similar to those recorded by Aluwong et al. (2013), who reported that the addition of probiotics stimulated the secretion of T3 and T4 through the physiological role of beneficial microbes directly to enhance the activity of corticotropin-releasing factors, which catalyze thyrotropin secretion. The positive effect of SC on T3 and T4 values in the current study could be explained via higher effective digestion of the diet due to increased activity of the intestinal enzymes or an increased absorptive ability of the intestinal mucosa as a result of morphological changes (Mountzouris et al. 2007), thus higher the nutrients available.

Antioxidant enzymes are important scavengers of free radicals which protect the body tissues from oxidative stress damage (Abdel-Moneim et al. 2020d, 2022a). In the present study, dietary supplements increased the activity of SOD. Noticeable improvement in oxidation state due to the inclusion of olives is due to some phenolic compounds (oleuropein, the main glycoside, and hydroxytyrosol) that have a role as potential antioxidants (Abd El-Moneim and Sabic 2019; Abd El-Moneim et al. 2019). Oke et al. (2017) reported similar results as they reported a significant difference in the performance of broiler chickens fed different doses of olive by-products in the feed, which led to improved performance and increased plasma SOD activity. El‐Deep et al. (2021) reported that probiotic feeding enhanced antioxidant properties (increased catalase and reduced MDA levels) and improved the antioxidative status in rabbits by up-regulating the antioxidative‐related genes.

In this study, SC or CA significantly enhanced growing rabbits’ plasma immunoglobulin IgM and IgG concentrations. These results are in agreement with those obtained by El-Shafei et al. (2019), who found that serum IgG levels of growing rabbits fed diets included with probiotics were higher compared to the control. The immunomodulatory impact of probiotics that stimulated IgM and IgG concentration of the monogastric animals has been reported (Abd El‐Hack et al. 2020; Abdel-Moneim et al. 2020c, 2022b, c). Furthermore, it can thus be deduced that citric acid or probiotics might improve immune statuses, which reduce weaning fatigue and improve rabbits’ performance (Li et al. 2009). The noticeable change in immunity may be due to the role of probiotics and organic acid in modifying the intestinal microbial community by increasing the beneficial microbes and decreasing the pathogenic microbes and their toxins. The reduction in pathogen load reduces their harmful effects on gut cells, enhances the immune response (Elbaz et al. 2022; Shokryazdan et al. 2017), and reduces the pro-inflammatory cytokines and therefore stimulates IgG production (Naqid et al. 2015).

The intestine is the main organ for the maintenance and production of animals by transferring the nutrients required. To study the health of the intestines, it is necessary to examine intestinal histomorphology (VH and CD), gastrointestinal tract morphology, and classification of microbial content (Paul et al. 2007). The results of the present study revealed a significant increase in the VH of rabbits fed a diet containing SC or CA. In line with our results, several reports revealed that probiotic or organic acid supplements boost VH in monogastric animals (El-Shafei et al. 2019; Elbaz et al. 2020; Elbaz 2021). Increasing the intestinal villi’s surface area increases the absorption rate, leading to enhancing the availability of nutrients and improving the animal’s immunity and performance (Abdel-Moneim et al. 2020c; Shehata et al. 2022).

Cecal microbial content in this study was greatly influenced by dietary supplements, as *Lactobacillus* count was increased and *E. coli* number was decreased in growing rabbits fed SC and AC. Consistent with our results, some reports found that the addition of organic acid or probiotic tends to have an increasing linear effect on *Lactobacillus* and reduce the pathogenic bacteria burden in the gut of many animals (Abdel-Moneim et al. 2022c; Shehata et al. 2021; Świątkiewicz et al. 2010). Also, some studies showed that organic acid linearly inhibited the *E. coli* population in the caecum of rabbits (Zhu et al. 2014). The positive effect of probiotics or organic acids on modifying the microbial content of the intestine may be due to their antimicrobial effect and the competitive exclusion mechanism between intestinal microbes on food and attachment sites at the surface of epithelial cells (Abd El‐Hack et al. 2020; Elbaz et al. 2021; Shehata et al. 2021). It can be concluded that *S. cerevisiae* or citric acid improves the host’s intestinal microbial balance and creates a favorable intestinal environment to support beneficial microbes and inhibit pathogenic microbes.

## Conclusion

Supplementing rabbit diets with *S. cerevisiae* or citric acid in combination with OC has been shown to enhance nutrient digestibility, growth performance, and carcass percentage. Additionally, these supplements improve intestinal structure, immune response, thyroid function, antioxidant levels, and the composition of gut microbiota in growing rabbits fed OC diets. *S. cerevisiae* supplementation was found to be more effective than citric acid in OC-based diets. These findings suggest that OC can be included in rabbit diets at levels of up to 20% alone or 25% when supplemented with *S. cerevisiae* and citric acid, offering a cost-effective source of fiber for optimal rabbit growth.

## Data Availability

All data generated or analyzed during this study are included in this published article.
